# Factors associated with psychological burden of breast cancer in women in Morocco: cross‑sectional study

**DOI:** 10.1186/s12905-023-02769-3

**Published:** 2023-11-10

**Authors:** Safiya Mahlaq, Laila Lahlou, Ismail Rammouz, Redouane Abouqal, Jihane Belayachi

**Affiliations:** 1 Laboratory of Biostatistics, Clinical Research and Epidemiology (LBRCE), Faculty of Medicine and Pharmacy of Rabat, Mohammed V University, Rabat, 10100 Morocco; 2 Faculty of Medicine and Pharmacy of Agadir, Ibn-Zohr University, Agadir, Morocco

**Keywords:** Breast cancer, Depression, Anxiety, Social Support, Morocco, Mental Health

## Abstract

**Background:**

Depression and anxiety are among the psychological diagnoses impacting individuals diagnosed with breast cancer. This study aims to estimate the prevalence, as well as the predictors, of anxiety and depression in women with breast cancer.

**Materials and methods:**

This was a cross-sectional, multi-center study conducted over an eight-month period among women with breast cancer in oncology centers in southern Morocco. Anxiety and depression were assessed using the validated Moroccan dialectal version of the Hospital Anxiety and Depression Scale (HADs). To identify the predictors of anxiety and depression in the study population. Multiple linear regression analyses were performed, including variables for which univariate analyses were significant with a p < 0.05 value. Statistical analyses were performed using Jamovi software version 2.2.3.

**Results:**

A total of 230 participant responses were collected. The prevalence of anxiety and depression was 77.4% and 62.6%, respectively. Multiple linear regression analysis revealed the following factors increased anxiety: being younger than 50 years old, not having studied beyond elementary school, having children aged between 10 and 18 and having TNM stage III and IV. The following factors decreased anxiety in patients with breast cancer: good physical functioning (Karnofsky score), satisfaction with social support and financial support. Regarding depression, the following factors decreased depression: good physical functioning (Karnofsky score), a minimum of 2.5 h per week of physical activity, active occupational status, satisfaction with social support and financial support. In contrast, the recurrence of breast cancer was an associated factor with increased depression.

**Conclusion:**

The prevalence of anxiety and depression in women with breast cancer is very high in our context. Therefore, routine screening tests for depression and anxiety as well as psychosocial management care are necessary for patients with breast cancer in Morocco.

## Background

Breast cancer constitutes a worldwide burden. According to data from Globocan (2020), breast cancer is the most commonly diagnosed cancer worldwide with an incidence of 24.5% of cancer cases in women and 11.7% in both sexes. It is the leading cause of death among cancer types (6.9%). In Morocco, it is the most common cancer in women (38.9%) among all types of cancer in both sexes (19.8%), and it is the 2nd cause of death by cancer (10.5%) [1].

Breast cancer and its treatment is an arduous ordeal for a woman’s body and mind. Indeed, the therapeutic management of breast cancer is associated with several treatment strategies however many are aggressive and responsible for physical, psychological and social consequences. Receiving the diagnosis of cancer can be emotionally challenging; the subsequent course of illness can further impact the psychological health of patients [2]. As a consequence, depression and anxiety can arise among those diagnosed with breast cancer [3]. A systematic review demonstrated that anxiety can persist not only throughout one’s course treatment but also after remission, regardless of the treatment protocol followed [4]. Considering anxiety and depression are the most common psychological disorders in women with breast cancer, there is great importance in focusing on these two psychological disorders.

In this setting, depression represents a challenge for both the patients who are diagnosed and for their caregivers. Such psychological implications impact both medical and economic aspects of oncological management [5]. A diagnosis of breast cancer with concomitant anxiety or depression has negative impacts on treatment compliance and leads to an increased risk of mortality. For many individuals, the term “cancer” is associated with a serious and very aggressive disease, so an anxious emotional reaction can ensue. Similarly, one’s perception of secondary losses can trigger depression. Such losses are multidimensional in terms of daily functioning, work, aesthetic, marital life and self-image and can lead to a disabling social isolation. Anxiety and depression additionally increase the risk of mortality in women with breast cancer, however data have shown that this risk is alleviated with antidepressants [6].

Nevertheless, depression is often not screened or treated adequately, resulting in great suffering and a considerable decrease in the quality of life of patients and their families. Indeed, isolation from society and even from the family has a harmful impact on patients, their families and their relationships. In contrast, satisfactory perceived social support has a beneficial effect on the physical and mental health of breast cancer patients [2].

In effect, breast cancer patients have a high risk of developing psychiatric disorders, depression and anxiety being the most common. Thus, improved screening of these disorders could lead to an enhanced sense of quality of life among patients diagnosed with breast cancer. The first step would be to have an effective screening strategy for anxiety and depression among patients at risk of psychological distress [7–9].

In Morocco there exists few data on this topic. According to a study conducted by the Moroccan National Institute of Oncology in Rabat, as it relates to the psychological care of patients diagnosed with breast cancer, the management strategy must be based on the detection of the principal factors associated with psychological distress [10]. Therefore, determining the prevalence of depression and anxiety, as well as the associated factors, can help public health officials design better prevention, screening and management plans. Consequently, this study was conducted to elucidate this epidemiological pattern.

This study had two objectives. The first was to estimate the prevalence of anxiety and depression among women with breast cancer undergoing treatment in southern Morocco. The second was to determine the sociodemographic and clinical predictors of these psychological disorders, including social and financial support. To our knowledge, this study is the first to examine the possible association between anxiety, depression and social support among the Moroccan breast cancer patient population.

## Patients and methods

### Study design

This was a multicenter cross-sectional study conducted in the Souss-Massa region of Morocco over an 8-month period (between June 2021 and January 2022). The inclusion criteria were as follows: female, over 18 years old, a confirmed diagnosis of breast cancer, started treatment for at least 8 weeks (chemotherapy, radiotherapy, surgery, targeted therapy) and attending oncology centers (public or private), which represents the first recourse for cancer patients in all regions of southern Morocco. Our exclusion criteria were as follows: having a previous follow-up for psychiatric disorders or having a previous diagnosis of primary cancer in a location other than the breast.

## Data collection and measurement instruments

### Sociodemographic and clinical data

Our questionnaire had two parts. The first focused on personal and sociodemographic data. The second focused on clinical data.

The personal and sociodemographic data included age, living area (rural, urban), marital status (single, married, divorced, widowed), educational level ( unschooled, primary, secondary, high school), occupational status (active, inactive, loss of work through disease), medical coverage (yes, no), socioeconomic status (low, average, high), minor children in charge (yes, no), age range of children ( no child, < 10 years, 10–18 years, > 18 years); family history of breast cancer (yes, no); moderate-intensity physical activity of 2.5 hours per week “walking or cycling / light sport / housework “: (yes, no); living conditions (with family, other), financial support received (yes, no).

Clinical characteristics included the following: time since diagnosis ( < = 1year, >1year); TNM cancer stage (0 ,I-II, III, IV); SBR cancer grade (I, II, III); histologic type of breast cancer (Invasive breast carcinoma, in situ carcinoma, other); molecular type (luminal A, luminal B, HER+ ,triple negative, unclassified); type of treatment received: chemotherapy (yes, no), radiotherapy (yes, no), hormonotherapy (yes, no), targeted therapy (yes, no), type of surgery (lumpectomy, mastectomy, not made), comorbidity (yes, no) and recurrence (yes, no).

The definition of educational level and socioeconomic level were based on the classification of the High Commission for Planning (HCP) of Morocco [11, 12]. Moderate physical activity was defined based on world health organization (WHO) reports [13]. The other sociodemographic and clinical variables were determined based on a literature review [2, 14–16]. to assess the physical functioning of the patient, the Karnofsky Performance Scale (KPS) was adopted.

In addition, anxiety, depression and perceived social support were evaluated by validated scales known as the Hospital Anxiety and Depression Scale (HADS) and The Social Support Questionnaire (SSQ6).

### Collection instrument

#### KPS (Karnofsky performance scale)

KPS is a unidimensional physical functioning scale used to obtain an overall measure of activity level. The level of functionality is assessed by a health professional as a percentage ranging from 100% (normal, no complaints and no symptom of disease) to 0% (death) [17].

#### HADs (Hospital anxiety and depression scale)

The Hospital Anxiety and Depression Scale was used to assess anxiety and depressive disorders. This is a self-reported scale developed by Zigmond and Snaith in 1983 to screen for anxiety and depressive disorders among hospitalized patients in nonpsychiatric settings. This questionnaire contains two subscales assessing anxiety and depression, each with seven items that were designed to measure emotional states independent of physical symptoms. It includes 14 items scored from 0 to 3. Seven items focus on anxiety (total A) and seven on the depressive dimension (total D), thus obtaining two scores (maximum score for each score = 21). The anxiety score is obtained by adding up the scores attributed to the anxiety questions. A score higher than or equal to 11 defines anxiety. The depression score is obtained by adding the scores attributed to the seven depression questions. A score higher than or equal to 11 defines depression [18]. The HADS has been recommended for use in cancer studies [19]. The study of the psychomotor properties of the Moroccan version of the HADS scale showed a good validity and reliability of the questionnaire adapted to the Moroccan dialect [20]. Cronbach’s alpha for anxiety and depression scores are 0.78 and 0.74, respectively, which demonstrates the good reliability of HADs used in our study.

#### SSQ6 (Social support questionnaire)

For perceived social support, The Social Support Questionnaire (SSQ) is a self-assessment scale whose objective is to assess the perception of availability and satisfaction with social support [21]. In this study, the six-item version, known as SSQ6, was used. Participants must first identify people who support them for each of the six statements. The questionnaire has a bifactorial structure that measures two dimensions of social support: availability and satisfaction [22]. This allows for a subjective assessment by the participant of the support provided through six questions in two parts. One is the availability of support (nature and number of people); the other is satisfaction with this support, assessed on a scale ranging from 1 (very dissatisfied) to 6 (very satisfied). In this study, social support was assessed using the Moroccan version of SSQ6 translated and used by Serhier (2017) [23]. Two total scores are then calculated, one of availability (N = number of people cited) and the other of satisfaction (S), which correspond, respectively to the sum of the score “number” (score N) and “satisfaction” (score S) obtained for each of the six items. The N score varies from 0 to 54 (0 corresponds to the absence of availability and 54 corresponds to the complete availability) and the S score varies from 6 to 36 (6 corresponds to the nonsatisfaction and 36 corresponds to total satisfaction) [21]. in this study, Cronbach’s alpha for satisfaction and availability scores are 0.94 and 0.93, respectively, which testifies to the very good reliability of SSQ6.

## Sampling and data collection procedure

The sample size calculation was performed using G*power software [24]. For a statistical power of 0.90, a mean effect size of 0.15 with 30 predictors, a necessary sample size of 226 was required to ensure a significance level of 0.05. The sample size was 230.

Patients were recruited at their treatment sessions and during follow-up visits according to eligibility criteria. Informed consent was obtained from participants after explanation of the objectives and voluntary nature of the study. A letter of consent was then signed. The average time required to complete the questionnaire was approximately 10–15 min.

### Statistical analysis

Data are presented as the mean ± standard deviation for variables with a normal distribution, and as the median and interquartile range (IQR) for variables with skewed distributions. Parametric or nonparametric tests were used for continuous variables as appropriate after the normality of the distribution was tested by the Shapiro-Wilk test. Statistical differences between two group for continuous variables were determined by Student’s t test or the Mann-Whitney U test. Comparison of differences among more group for continuous variables was carried out by the one-way ANOVA or Kruskal-Wallis test and Pearson’s correlation or spearman’s correlation was used to verify the association between two continuous variables. As our study is exploratory, Variables with P value lower than 0.05 in the univariate analysis were tested in the multivariate analysis. Multivariate analysis was performed using linear regression models. A two-tailed P value < 0.05 was considered statistically significant. Statistical analyses were carried out using Jamovi software version 2.2.3 [25, 26].

## Results

### Socio-demographic and clinical characteristics of participants

A total of 230 women with breast cancer eligible for the study were included. Of these patients, 78.3% were recruited from the public sector and 21.7% from the private sector.

The mean age of the patients was 49.2 ± 10.5 years, ranging from 27 to 80 years. Two-thirds (66%) of patients were unschooled, and more than half (57%) were from urban areas. Two hundred patients (87%) had a low socioeconomic status, and one hundred and sixty-one (70%) had received financial support from other sources. In terms of occupational status, the majority (72.2%) had no professional activity. Almost all (93.9%) of the patients lived with family, and slightly over two-third (67.8%) were married. Approximately half (49.5%) had minor children they cared for. Twenty (8.7%) study participants engaged in moderate physical activity for at least 2.5 h per week, while the remaining number of participants (91.3%) did less. A family history of cancer has been reported in eighty-nine cases (38.7%). (Table 1)

**Table 1:** Description of socio-demographic and personal information:

Variable	All participants N (%)	Description			
		Depression	P	Anxiety	P
Health sector:					
Public Private	180(78.3) 50(21.7)	12.14 ± 4.06 12.40 ± 4.54	0.702	14.26 ± 4.07 14.16 ± 4.35	0.878
Living area					
Rural Urban	99(43) 131(57)	12.22 ± 4.01 12.18 ± 4.28	0.944	14.70 ± 3.85 13.89 ± 4.30	0.144
Age					
< 50 years ≥ 50 years	49.2 ± 10.5 126(54.8) 104(45.2)	12.13 ± 4.05 12.28 ± 4.31	0.795	14.90 ± 3.73 13.43 ± 4.44	0.008*
Level of education					
unschooled Primary Secondary High school	150(65.2) 46(20) 17(7.4) 17(7.4)	12.35 ± 3.93 13.04 ± 4.42 10.06 ± 4.92 10.71 ± 3.92	0.031**	14.27 ± 4.15 15.56 ± 3.84 12.29 ± 4.15 12.35 ± 3.39	0.006**
Medical coverage					
Yes No	219(95.2) 11(4.8)	13.18 ± 5.47 12.15 ± 4.09	0.424	13.73 ± 5.83 14.26 ± 4.04	0.769
Marital status					
married Widowed Divorced single	156(67.8) 19(8.3) 22(9.6) 33(14.3)	11.85 ± 3.95 12.00 ± 4.29 14.18 ± 4.24 12.64 ± 4.77	0.089	13.95 ± 4.01 13.00 ± 5.27 16.36 ± 3.20 14.88 ± 4.08	0.015**
Living conditions					
Family Other	216(93.9) 14(6.1)	12.01 ± 4.12 15.07 ± 3.89	0.007*	14.06 ± 4.11 17.07 ± 3.38	0.008*
Socioeconomic level					
Low average High	200(87) 18(7. 8) 12(5.2)	12.06 ± 5.52 10.58 ± 3.63 12.31 ± 4.05	0.375	14.06 ± 4.38 13.08 ± 3.58 14.32 ± 4.14	0.589
Occupational status					
Active Inactive Loss of work	11(4.8) 166(72.2) 53(23)	12.18 ± 5.02 12.11 ± 4.02 12.47 ± 4.49	0.863	13.27 ± 3.87 14.17 ± 4.18 14.66 ± 4.03	0.549
Financial support received					
Yes No	161(70) 69(30)	11.83 ± 4.16 13.06 ± 4.06	0.040*	13.88 ± 4.05 15.09 ± 4.20	0.041*
Minor children in charge					
Yes No	114(49.6) 116(50.4)	12.62 ± 3.95 11.78 ± 4.34	0.127	15.06 ± 3.77 13.43 ± 4.30	0.003*
Age of children					
No child < 10years 10–18 years Major	56(24.4) 44(19.1) 70(30.4) 60(26.1)	12.50 ± 4.37 12.34 ± 4.21 12.80 ± 3.79 11.12 ± 4.23	0.118	14.77 ± 3.89 14.77 ± 4.19 15.24 ± 3.51 12.18 ± 4.33	< 0.001**
Comorbidity					
Yes No	78(33.9) 152(66.1)	12.72 ± 4.40 11.93 ± 4.02	0.177	14.26 ± 4.30 14.23 ± 4.04	0.964
Family history of cancer					
Yes No	89(38.7) 141(61.3)	12.43 ± 4.32 12.06 ± 4.07	0.512	14.10 ± 4.17 14.33 ± 4.10	0.688
Physical activity					
Yes No	20(8.7) 210(91.3)	8.90 ± 2.59 12.51 ± 4.15	< 0.001*	12.00 ± 3.11 14.45 ± 4.15	0.011*

*T-student test, **The one-way ANOVA test., N: number, %: percentage

Regarding the clinical characteristics of the study participants, 74.3% of patients had been diagnosed for less than one year. Almost half (42.5%) of patients were diagnosed with TNM stage III, and 18.1% were diagnosed at a metastatic stage (TNM stage V). Invasive breast carcinoma (IBC) was the histological type identified in 96.5% of the patients. According to molecular classification, luminal type A was the most frequent, as presented in 40% of patient samples. The grade of carcinoma most identified (in 54.3% of patient cases) was SBR III. As for the type of treatment received by patients, 99.1% had chemotherapy, 76.5% had radiotherapy, 53.5% had hormone therapy, 43.5% had targeted therapy, 68.3% had mastectomy, so fifteen patients (6.5%) had a recurrence in breast cancer. (Table 2)

**Table 2:** Description of clinical data

Variable	All participants N (%)	Description			
		Depression	P	Anxiety	P
Time since diagnosis					
≤ 1year > 1year	10[6-13.75] 171(74.3) 59(25.7)	- 11.97 ± 3.86 12.86 ± 4.91	0.090 0.208	- 13.88 ± 4.01 15.27 ± 4.29	0.009*** 0.025*
Histological type					
Invasive breast carcinoma In situ carcinoma Other	222(96.5) 6(2.6) 2(0.9)	12.24 ± 4.18 10.17 ± 2.86 13.50 ± 4.95	0.440	14.32 ± 4.11 13.00 ± 4.00 9.00 ± 2.83	0.146
Molecular type					
Luminal A Luminal B HER+ Triple negative Non-class	92(40) 71(30.9) 30(13) 31(13.5) 6(2.6)	12.35 ± 4.44 11.14 ± 3.51 13.10 ± 4.33 13.48 ± 4.27 11.33 ± 3.33	0.052	14.20 ± 4.37 13.37 ± 3.86 15.27 ± 4.02 15.45 ± 3.79 13.83 ± 3.92	0.098
SBR grade					
Grade I Grade II Grade III	5(2.2) 125(54.3) 100(43.5)	10.80 ± 4.66 11.91 ± 3.91 12.63 ± 4.43	0.329	11.20 ± 5.76 14.06 ± 4.19 14.62 ± 3.91	0.149
TNM Stage					
Stage I-II Stage III Stage IV	87(39.3) 94(42.5) 40(18.1)	10.86 ± 4.22 12.33 ± 3.72 14.55 ± 3.88	< 0.001**	12.75 ± 3.89 14.57 ± 3.94 16.45 ± 3.61	< 0.001**
Chemotherapy					
Yes No	228(99.1) 2(0.9)	12.19 ± 4.16 13.50 ± 4.95	0.658	14.28 ± 4.11 9.00 ± 2.83	0.071
Hormonotherapy					
Yes No	123(53.5) 107(46.5)	11.85 ± 3.88 12.61 ± 4.44	0.171	14.05 ± 3.84 14.46 ± 4.43	0.459
Targeted therapy					
Yes No	100(43.5) 130(56.5)	11.63 ± 3.87 12.64 ± 4.33	0.068	13.76 ± 3.87 14.61 ± 4.29	0.122
Radiotherapy					
Yes No	176(76.5) 54(23.5)	12.22 ± 4.28 12.13 ± 3.79	0.887	14.20 ± 4.16 14.37 ± 4.04	0.790
Surgery					
Lumpectomy Mastectomy No	47(20.4) 157(68.3) 26(11.3)	10.94 ± 4.77 12.40 ± 3.89 13.27 ± 4.24	0.040**	13.06 ± 4.06 14.37 ± 4.03 15.58 ± 4.41	0.034**
Recurrence					
Yes No	15(6.5) 215(93.5)	17.27 ± 3.63 11.85 ± 3.97	< 0.001*	17.73 ± 4.03 13.99 ± 4.03	< 0.001*

*T-student test, **The one-way ANOVA test. ***spearman’s correlation, N: Number, %: Percentage.

### Perceived social support and Karnofsky score

The mean Karnofsky score was 70.5 ± 12.2, ranging from 40 (disabled physical state) to 90 (normal activity). The median social support availability score (SSQ6) was 8 (6;11), while the median social support satisfaction score was 24 (19;30). (Table 3)

### Prevalence of anxiety and depression

The prevalence of depression demonstrated in this study was 62.6%, with a mean score of 12.2 ± 4.12. The prevalence of anxiety was 77.4%, with a mean score of 14.2 ± 4.12. (Table 3)

**Table 3:** Description of measurement scales (HADs/SSQ6/Karnofsky)

Score	Items	Possible interval	Min-Max scores	All patients	Depression		Anxiety		Cronbach’sα
					Spearman Correlation	P	Spearman Correlation	P	
Social support									
Satisfaction Availability	6 6	6–36 0–54	6–36 0–39	8 (6;11) 24 (19;30)	-0.624 -0.495	**< 0.001** **< 0.001**	-0.530 -0.414	**< 0.001** **< 0.001**	0.94 0.93
Karnofsky score		0-100	40–90	70.5 ± 12.2	-0.489	**< 0.001**	-0.352	**< 0.001**	
HADs									
Anxiety Presence ≤ 11 Absence > 11 Depression Presence ≤ 11 Absence > 11	7 7	0–21 0–21	4–21 4–21	14.2 ± 4.12 178(77.4) 52(22.6) 12.2 ± 4.12 144(62.6) 86(37.4)	- -	- -	- -	- -	0.74 0.78

HADs: hospital anxiety and Depression scale, SSQ6: Social Support Questionnaire.

### Predictive factors associated with anxiety and depression in multivariate analysis

In this study, independent variables included in the multiple linear regression model were chosen according to the significant univariate regression p value < 0.05. (See Table 4)

**Table 4:** Univariate analysis of factors associated with anxiety and depression

Variable	Univariate analysis					
	Depression			Anxiety		
	β	95% CI	P	β	95% CI	P
Age < 50	-0.144	-1.23, 0.94	0.795	1.472	0.41, 2.53	**0.007**
Health sector: Private /Public	0.256	-1.06, 1.57	0.702	-0.101	-1.40, 1.20	0.878
Living area : Urban /Rural	-0.039	-1.13, 1.05	0.944	0.548	-1.88, 0.27	0.144
Level of education						
unschooled Primary Secondary High school	1.647 2.338 -0.647 Ref	-0.42, 3.72 0.04, 4.63 -3.42, 2.13	0.118 **0.046** 0.646	1.914 3.212 -0.059 Ref	-0.12, 3.95 0.95, 5.47 -2.79, -2.67	0.065 **0.006** 0.966
Medical coverage	-1.031	-3.57, 1.50	0.424	0.538	-1.98, 3.05	0.674
Marital status						
married Widowed Divorced single	Ref 0.147 2.329 0.784	-1.83, 2.12 0.48, 4.18 -0.77, 2.34	0.883 **0.014** 0.323	Ref -0.955 2.409 0.924	-2.90, 0.99 0.58, 4.23 -0.61, 2.46	0.335 **0.010** 0.237
Living conditions: Family/Other	-3.058	-5.29, -0.83	**0.007**	-3.016	-5.23, -0.80	**0.008**
Socioeconomic level						
Low average High	Ref -0.254 -1.727	-2.27, 1.76 -4.16, 0.71	0.804 0.164	Ref -0.269 -1.242	-2.27, -1.73 -3.66, -1.18	0.791 0.313
Occupational status						
Inactive Loss of work Active	0.357 0.067 Ref	-0.94, 1.66 -2.49, 2.63	0.588 0.959	0.896 1.388 Ref	-1.64, -3.43 -1.31, -4.08	0.487 0.312
Financial support received	-1.226	-2.40, -0.05	**0.040**	-1.211	-2.37, -0.05	**0.041**
Minor children in charge	0.838	-0.24, 1.92	0.127	1.630	0.58, 2.68	**0.003**
Age of children						
No child < 10 years 10–18 years major	1.383 1.224 1.683 Ref	-0.13, 2.90 -0.39, 2.84 0.25, 3.12	0.073 0.137 **0.022**	2.585 2.589 3.060 Ref	1.13, 4.03 1.04, 4.14 1.69, 4.43	**< 0.001** **0.001** **< 0.001**
Comorbidity	0.784	-0.36, 1.92	0.177	0.026	-1.11, 1.16	0.964
Family history of cancer	0.370	-0.74, 1.48	0.512	-0.225	-1.33, -0.88	0.688
Physical activity	-3.614	-5.48, -1.75	**< 0.001**	-2.452	-4.33, -0.57	**0.011**
Time since diagnosis	0.047	0.003, 0.09	**0.036**	0.055	0.01, 0.10	**0.014**
Histological type						
Infiltrating carcinoma In situ carcinoma Other	2.077 3.333 Ref	-1.32, 5.47 -3.37, 10.03	0.229 0.328	5.320 4.000 Ref	-0.43, 11.07 -2.61, 10.61	0.069 0.234
Molecular type						
Luminal A Luminal B HER+ Triple negative Non-class	1.014 -0.192 1.767 2.150 Ref	-2.40, 4.43 -3.64, 3.25 -1.86, 5.39 -1.46, 5.76	0.559 0.912 0.338 0.242	0.362 -0.467 1.433 1.618 Ref	-3.03, 3.76 -3.89, 2.96 -2.17, 5.04 -1.97, 5.21	0.834 0.788 0.434 0.376
SBR grade						
Grade I Grade II Grade III	Ref 1.112 1.830	-2.62, 4.85 -1.92, 5.58	0.558 0.338	Ref 2.856 3.420	-0.83, 6.55 -0.29, 7.13	0.129 0.071
TNM Stage						
Stage I-II Stage III Stage IV	Ref 1.468 3.688	- 0.31, 2.63 2.20, 5.18	**0.013** **< 0.001**	Ref 1.827 3.703	0.69, 2.96 2.25, 5.16	**0.002** **< 0.001**
Chemotherapy	-1.311	-7.14, 4.52	0.658	5.285	-0.46, 11.03	0.071
Hormonotherapy	-0.762	-1.84, 0.32	0.166	0.409	-0.67, 1.48	0.454
Targeted therapy	-1.008	-2.09, 0.08	0.068	-0.848	-1.92, 0.23	0.122
Radiotherapy	0.092	-1.19, 1.37	0.887	-0.172	-1.44, 1.09	0.790
Surgery						
Lumpectomy Mastectomy No	Ref 1.465 2.333	0.11, 2.81 0.35, 4.32	**0.034** **0.021**	Ref 1.306 2.513	-0.03, 2.64 0.55, 4.48	0.056 **0.012**
Recurrence	5.420	3.34, 7.50	**< 0.001**	3.738	1.62, 5.86	**< 0.001**
Social support						
Satisfaction Availability	-0.348 -0.258	-0.40, -0.29 -0.33, -0.18	**< 0.001** **< 0.001**	-0.293 -0.221	-0.35, -0.23 -0.30, -0.15	**< 0.001** **< 0.001**
Karnofsky	-0.179	-0.22, -0.14	**< 0.001**	-0.127	-0.17, -0.09	**< 0.001**

β value: standardized beta-coefficient, CI: confidence interval, Ref: Reference level.

Multiple linear regression analysis revealed that factors significantly associated with high level of anxiety in breast cancer patients were: age less than 50 years (β = 1.83, 95% CI: 0.78, 2.89, p < 0.001), primary education (β = 2.79, 95% CI: 1.01, 4.57, p = 0.002), having minor children between the ages of 10 and 18 years (β = 1.43, 95% CI: 0.22, 2.65, p = 0.021), TNM stage III (β = 1.22, 95% CI: 0.21, 2.22, p = 0.018), and TNM stage IV (β = 1.90, 95% CI: 0.38, 3.40, p = 0.014). The following factors were associated with a low level of anxiety: financial support received (β=-1.11, 95% CI: -2.04, -0.19, p = 0.019), social support satisfaction (β=-0.17, 95% CI: -0.25, -0.10, p < 0.001), and physical functioning of the patient (the Karnofsky score) (β=-0.08, 95% CI: -0.12, -0.03, p < 0.001). (See Table 5)

Regarding depression in breast cancer patients, multiple linear regression analysis demonstrated that recurrence of breast cancer was positively associated with a high level of depression (β = 2.26, 95%CI: 0.59, 3.93, p = 0.008), while the following factors were negatively associated with high levels of depression: physical activity of at least 2.5 h per week (β=-1.91, 95% CI: -3.25, -0.57, p = 0.005), physical functioning based on the Karnofsky score ( β=-0.11, 95% CI: -0.15, -0.08, p < 0.001), social support satisfaction (β=-0.24, 95% CI: -0.31, -0.17, p < 0.001) and financial support received (β=-0.86, 95% CI: -1.71, -0.01, p = 0.048), (see Table 5).

**Table 5:** Multiple linear regression analysis of associated factors with anxiety and depression

Variable	Multivariate analysis					
	Depression			Anxiety		
	β	95% CI	P	β	95% CI	P
Age < 50				1.835	0.78, 2.89	< 0.001
Education Level (Primary)				2.792	1.01, 4.57	0.002
Financial support received	-0.860	-1.71, -0.01	0.048	-1.115	-2.04, -0.19	0.019
Age of children (10–18 years)				1.433	0.22, 2.65	0.021
Physical activity	-1.909	-3.25, -0.57	0.005			
Satisfaction of social support	-0.245	-0.31, -0.17	< 0.001	-0.172	-0.25, -0.10	< 0.001
Karnofsky	-0.114	-0.15, -0.08	< 0.001	-0.076	-0.12, -0.03	< 0.001
TNM Stage						
Stage III Stage IV				1.216 1.895	0.21, 2.22 0.38, 3.40	0.018 0.014
Recurrence	2.263	0.59, 3.93	0.008			

β value: standardized beta-coefficient, CI: confidence interval.

## Discussion

The diagnosis and treatment of breast cancer can impact one’s life in many ways, including psychosocially. This is supported by a recent study that found that breast cancer patients had the highest prevalence of anxiety and depression among all other cancer patients [27].

To our knowledge, this is the first study in Morocco that has examined the impact of social support on anxiety and depression among women with breast cancer.

The results of this study allow for a more comprehensive understanding of predictive factors as they relate to anxiety and depression in women with breast cancer. This may provide a better management protocol to treat and even prevent psychological disorders in this population.

### Prevalence of anxiety and depression

One of the main aims of our study was to estimate the prevalence of anxiety and depression in breast cancer patients. In line with national and international studies, our results indicate that a high number of patients suffer from these disorders.

The prevalence of anxiety disorders in our study was 77.4%; this is similar to a study conducted in Bolenga Liboko in Congo which found that 74% of their patients with breast cancer had anxiety [28]. A study in China found this prevalence to be 73.26% [29], 66% in a recent study in Morocco [30], and 65% and 48% in Tunisia in research study in 2016 and 2018, respectively [31, 32].

The prevalence of depression in this study was 62.6%. Among patients with breast cancer, other studies found the following prevalence of depression: 59% in a recent study in Morocco [30], 61% in a study in Malaysia [33], 51.5% in a study in Tunisia in 2016 [32], and 37% in the work of Faten in 2018 [31]. It is higher in other studies; for instance, 70.44% in a study in China [34] and 79.33% in Congo [28]. In general, the prevalence of anxio-depressive disorders among women with breast cancer, reported by a recent study in Morocco, is 86.67% [35], while in other countries [9, 33, 36, 37], the rate of anxiety and depression do not exceed 31.7% and 22%, respectively. This variation may be justified by the difference in screening practices and treatment approaches as well as by cultural and socioeconomic differences between countries. In a meta-analysis entitled “Global prevalence of depression in breast cancer patients,” which consisted of 72 studies in 30 countries, subgroup analysis based on WHO regions showed that the combined prevalence of anxiety and depression was lowest in America (25.1%), Europe (27.2%) and the Western Pacific (29.9%) and highest in EMRO (51.5%) [38].

### Predictive factors for anxiety and depression

Screening for psychological distress and its complications, more specifically anxiety and depression, can be challenging. Additionally, living with an undiagnosed and untreated mental health disorder can further exacerbate one’s underlying distress of living with a new diagnosis. Considering this, it may be helpful to identify predictive factors of anxiety and depression in women with breast cancer as a means to enhance screening and treatment efforts.

The results of this multiple linear regression analysis revealed factors that were associated with anxiety and depression in breast cancer patients. These factors were principally sociodemographic and clinical.

Among sociodemographic factors, having minor children in charge was a risk factor for anxiety in the study population; women with adolescents aged 10 to 18 years had higher levels of anxiety (β = 1.43, 95% CI: 0.22, 2.65, p = 0.021). Other studies have confirmed these results. For example, Park (2018) found that being a mother of small children was a factor of psychological distress for young women with breast cancer [39]. One explanation for this association is that women diagnosed with breast cancer fear that if they were to die, their children may be left alone [40]. Additionally, Dunn reported that the fear of not being able to raise children can disrupt the mother-child relationship and further impact parenthood already threatened by the constraints of the disease [41]. Osborne and all concluded: “She sometimes felt guilty for not being fully present for these children; as a result of the decrease in her energy level; She could not play her role as a mother according to her wishes and this was difficult for her to accept, as she did not want to disappoint them” [45].

According to our results, young patients under the age of 50 had higher anxiety scores (14.90 ± 3.73) than older ones. Namely, younger patient age (< 50 years old) was positively associated with anxiety (β = 1.83, 95% CI: 0.78, 2.89, p < 0.001), which was predictive of anxiety. This is consistent with a study conducted in Australia, reporting that younger age at diagnosis (< 50 years) is a risk factor for anxiety and depression [42]. Similarly, younger patients at working age were more anxious but less depressed (p < 0.001) [37]. Another German study in 2022 also indicated that patients under 50 years old show higher levels of anxiety (β = 1.08 (0.03, 2.12), p = 0.04), while older patients are often less affected [43].

A primary level of education was a risk factor for developing anxiety when compared to higher educational levels (β = 2.79, 95% CI: 1.01, 4.57, p = 0.002). Similarly, another study concluded that education level is a factor associated with anxiety and that women with low levels of education were four times more likely to be anxious than those with a higher level of education [44]. Other researchers have also demonstrated this finding [9].

Physical exercise was also a protective factor against depression. Women who engaged in moderate physical exercise were less likely to have depression disorders than others, even posttreatments. Physical exercise had significant positive correlations with self-esteem, overall health and quality of life (physical, emotional, cognitive and social aspects) [3]. Exercise of at least 2.5 h per week was negatively correlated with depressive symptoms(r =-0.82) [45]. This supports the psychological protection that exercise has against depression among women with breast cancer. Considering this, perhaps exercise can be among a recommended lifestyle practice for cancer survivors to achieve greater self-esteem, a better quality of life and a decrease in depression symptoms. These findings are reinforced by the results of our study, which showed that moderate physical activity of at least 2.5 h per week reduced the depression score (β=-1.91, 95% CI: -3.25, -0.57, p = 0.005).

Social support is an additional determinant of psychological and mental well-being. Our study described this support in two dimensions: availability and satisfaction. Regarding the social support scores, the median social support availability score was 8 (6;11), while the median social support satisfaction score was 24 (19;30). There was a significant negative association between social support satisfaction and anxiety (β=-0.17, 95% CI: -0.25, -0.10, p < 0.001) and depression (β=-0.24, 95% CI: -0.31, -0.17, p < 0.001); therefore, the scores for anxiety and depression decreased as social support satisfaction score increased. For social support availability, no relationship was observed.

Consistent with these findings, one study found associations between satisfaction with social support and improved global quality of life and other domains of quality of life, including psychological domains (p = 0.004) [46]. Another study observed a significant negative correlation between anxiety and social support (r = -0.334, p < 0.01) [34]. Social support was again found to be a protective factor against anxiety and depression in breast cancer survivors [37]. Similarly, according to a recent study carried in Addis Ababa in 2019, there was a statistically significant association (P = 0.027) between social support and depression [47]. Another study in Taiwan in 2017, concluded that social support was a protective factor against depression (aOR = 0.87, 95% CI [0.78–0.98]) [48]. As demonstrated by these several studies, low social support is among the risk factors for anxiety and depression in women with breast cancer [42].

The availability of financial resources represented an assurance to face the expenses of life and care in cancer patients; in contrast, the lack of financial resources was a source of stress. A Moroccan study found that financial difficulties were a risk factor for psychological disorders (anxiety and depression) [OR = 2.09; p = 0.037] [10]. Our study similarly illustrated that financial support is a protective factor against anxiety (β=-1.11, 95% CI: -2.04, -0.19, p = 0.019) and depression (β=-0.86, 95% CI: -1.71, -0.01, p = 0.048). Such findings in Morocco are in accord with another study that concluded that patients with less financial support were 3 to 4 times more likely to have depressive disorders [36].

Our study concluded that physical functioning assessed by the Karnofsky score was negatively associated with anxiety (β=-0.08, 95%CI: -0.12, -0.03, p < 0.001) and depression (β=-0.11, 95% CI: -0.15, -0.08, p < 0.001), so that the better the physical condition was, the lower anxiety and depression scores were; consequently, the patient was protected against anxiety and depression. These results are supported by another study that concluded that better physical health was associated with decreased anxiety and depression [37]. Notably, another study reported that psychological distress (depression and anxiety disorders) was associated with poorer physical function [49]. There are also data to support that patients with more physical symptoms were 3.8 times (95% CI [1.20, 11.76]) more likely to develop a depressive disorder than those who had normal activity without any or some physical symptoms [9].

The stage of cancer at the time of diagnosis was another risk factor for the development of anxiety. The present study confirmed that advanced stages of breast cancer were predictive of anxiety: TNM III (β = 1.22, 95%CI: 0.21, 2.22, p = 0.018) and IV (β = 1.90, 95%CI: 0.38, 3.40, p = 0.014). Similarly, a study conducted by Aquil (2021) found that advanced cancer stage was associated with higher mental disorders (< 0.001) [30]. Moreover, various studies have reported that cancer patients with distant metastases had more psychological distress than patients with localized or locoregional disease [50, 51].

In a German study performed in 2020, time since diagnosis, advanced stage, treatments, and having children were not significant risk factors for depression; nonetheless, recurrence was associated with depression [52]. This finding is similarly demonstrated by our study, which found that recurrence was positively associated with depression (β = 2.26, 95%CI: 0.59, 3.93, p = 0.008). Additionally, a Chinese meta-analysis study in 2020 found that depression was associated with cancer recurrence (β = 1.24, 95%CI: 1.07,1.43) [53].

The study findings provided clinical recommendations to guide a strategic approach to the multidisciplinary management care of breast cancer patients. The high prevalence of anxiety and depression in our setting highlights the crucial need to implement systematic screening for the psychological burden associated with breast cancer. Moreover, it underscores the urgency of implementing a tailored care management approach that addresses the specific psychological needs of these patients.

Healthcare decision-makers are also called upon to adopt new protocols for oncology management care that integrate supportive care in oncology, in particular psychosocial care such as support groups, social support in a family approach, and psychological accompaniment of patients and their caregivers. Furthermore, promoting health education is crucial to encouraging patients to adopt a healthier lifestyle, including moderate physical activity. The discipline of psycho-oncology should also be encouraged to provide caregivers with the professional skills they need for the psychological management of breast cancer patients.

### Strengths and limitations of the study

One strength of our study is that it was conducted at several sites across oncology centers in both the public and private sectors. The latter covers the entire southern region, which encompasses four out of twelve regions of Morocco, according to the territorial division of the kingdom. Another strength of our study is that it was the first study in this region to estimate the prevalence of anxiety and depression and to investigate the social, demographic, and clinical factors associated with them. Additionally, this study is the first in Morocco to investigate social support as a factor associated with anxiety and depression in breast cancer patients.

This study also had limitations. One limitation is that due to our limited sample size, the odds ratios were wide. Additionally, there are other factors that may be associated with anxiety and depression that were not included in this study, such as personality traits, resilience parameters, degree of religiosity, or environmental factors such as care circuit and ease of access to care. However, the results obtained may provide a solid basis for future studies that will further explore this subject.

## Conclusion

The prevalence of anxiety and depression in women with breast cancer in southern Morocco was 77.4% and 62.6%, respectively. Factors that increased the risk of depression and anxiety among breast cancer patients included being under 50 years of age, having minor children, having a low level of education, being diagnosed at an advanced cancer stage and having a recurrence of breast cancer. Such increasing factors may be useful in a clinical setting to help screen women diagnosed with breast cancer who are at a higher risk of developing depression or anxiety.

The decrease factors identified were as follows: good physical functioning, moderate physical activity of at least 2.5 h per week, adequate financial resources, and social support. Such risk factors may be useful in a clinical setting to help screen women diagnosed. Such factors may be helpful to introduce as possible preventive tools to help mitigate symptoms of depression and anxiety among women diagnosed with breast cancer.

In addition to increasing screening initiatives for anxiety and depression among women diagnosed with breast cancer, increasing access to mental healthcare and psycho-oncology services may also provide a great benefit to this patient population.

## Data Availability

The datasets used and/or analyzed during the current study are available from the corresponding author upon reasonable request.

## Abbreviations

EMRO: the Eastern Mediterranean Region
HADs: Hospital Anxiety and Depression Scale
HER+: Human Epidermal growth factor Receptor Positive
HCP: High Commission for Planning of Morocco
IQR: Interquartile Range
IBC: Invasive Breast Carcinoma
SSQ6: Social Support Questionnaire
KPS: Karnofsky Performance Scale
STROBE: Strengthening the Reporting of Observational Studies in Epidemiology
SBR cancer grade: Scarff, Bloom et Richardson cancer grade
TNM stage: Tumour, Node and Metastasis stage
WHO: World Health Organization
