# Correction: Factors associated with psychological burden of breast cancer in women in Morocco: cross‑sectional study

**DOI:** 10.1186/s12905-024-02896-5

**Published:** 2024-01-23

**Authors:** Safiya Mahlaq, Laila Lahlou, Ismail Rammouz, Redouane Abouqal, Jihane Belayachi

**Affiliations:** 1 Laboratory of Biostatistics, Clinical Research and Epidemiology (LBRCE), Faculty of Medicine and Pharmacy of Rabat, Mohammed V University, Rabat, 10100 Morocco; 2 Faculty of Medicine and Pharmacy of Agadir, Ibn-Zohr University, Agadir, Morocco

**BMC Women’s Health (2023) 23:590**

10.1186/s12905-023-02769-3

Following publication of t he original article [1], the author name “Safiya Mahlaq” was incorrectly written as “Safiya Mahalq” and author name has been corrected.

The original article has been corrected.
